# Placement of *Spongiphora lewisi* de Bormans, 1903, an enigmatic earwig species of Spongiphoridae (Insecta, Dermaptera), as revealed by integrative taxonomy

**DOI:** 10.3897/zookeys.1275.182541

**Published:** 2026-04-02

**Authors:** Yoshitaka Kamimura, Meliana Gusti

**Affiliations:** 1 Department of Biology, Keio University, Kanagawa, 223-8521, Japan Tokyo Metropolitan University Tokyo Japan; 2 Department of Biological Sciences, Graduate School of Science, Tokyo Metropolitan University, Tokyo, 192-0397, Japan Keio University Kanagawa Japan

**Keywords:** Female genitalia, mycophily, *
Nesogaster
*, Nesogastrinae, ovarian fertilization, Spongiphorinae, *
Spongovostox
*, tegminal keel

## Abstract

*Spongiphora lewisi* de Bormans, 1903 is a large spongiphorid earwig, known from Japan, China, Laos, and Vietnam. Although many previous authors have placed this species under *Nesogaster* (Nesogastrinae) focusing on their developed tegminal (forewing) keels, the taxonomy of this species, which shows specific mycophily to fruiting bodies of *Cryptoporus volvatus*, requires further scrutiny. In this study, multiple lines of evidence for placement of this species under *Spongovostox* (Spongiphorinae) are provided. The morphologies of the male and female genitalia (the latter reported for the first time) and DNA barcodes indicate close affinities of *Sp. lewisi* to two spongiphorine genera, *Spongovostox* and *Marava*. Embryonic development in this species begins within the ovaries, indicating ovarian fertilization, a phenomenon previously documented in several species of Spongiphorinae, Labiinae, and Sparattinae, but not in Nesogastrinae. Although the tegminal keel structures of *Spongiphora lewisi* resemble those of true *Nesogaster*, their different orientations support reinstating the combination *Spongovostox lewisi* (de Bormans, 1903). Our integrative taxonomy analysis reveals instability in the subfamilial classification of Spongiphoridae, which relies heavily on a limited set of traits prone to convergent evolution. A revised key for Spongiphoridae subfamilies is also presented, and a new mainland Sumatra record of *Auchenomus javanus* (Sparattinae) is reported.

## Introduction

Dermaptera, a polyneopteran order commonly known as earwigs, includes more than 2,000 extant species (Grimaldi and Engel 2005; Zhang 2013; Haas 2018; Hopkins et al. 2025). Spongiphoridae (= Labiidae) is among the most speciose families of this order, consisting of approximately 500 species (Hopkins et al. 2025). Characterized by a single penis in males, Spongiphoridae is classified within the parvorder Eudermaptera (Engel and Haas 2007; Kamimura 2025). Multiple lines of both morphological and molecular evidence support the monophyly of Eudermaptera (Haas 1995; Haas and Kukalová-Peck 2001; Jarvis et al. 2005; Kočárek et al. 2013; Naegle et al. 2016; Wipfler et al. 2020; Shimizu and Machida 2024; Kamimura 2025). However, unlike the other four families in this parvorder (according to the system of Kamimura 2025: Forficulidae and Chelisochidae with distinct leg morphologies, and Arixeniidae and Hemimeridae with specialized morphological adaptations for mammalian association), Spongiphoridae lacks a clear synapomorphy. Consequently, its potential paraphyly or even polyphyly has been suggested by several studies (Jarvis et al. 2005; Biliński et al. 2014). Subfamilial classifications within Spongiphoridae are similarly unstable, relying on a limited set of traits and varying across researchers (Table 1; Steinmann 1990; Srivastava 1996a).

**Table 1.** Classification systems for the family Spongiphoridae Verhoeff, 1902. Generic names shown in bold indicate genera causing major generic-level discrepancies between the two systems.

Steinmann (1990)*: followed in the present study	Srivastava (1996a, 2013)**
Pericominae Burr, 1911a	Pericominae Burr, 1911а
(*Pericomus* Burr, 1911a; *Parapericomus*, Ramamurthi, 1967)	(*Pericomus* Burr, 1911a; *Parapericomus*, Ramamurthi, 1967)
Nesogastrinae Verhoeff, 1902	Nesogastrinae Verhoeff, 1902
(*Nesogaster* Verhoeff, 1902)	(*Nesogaster* Verhoeff, 1902)
Ramamurthiinae*** Steinmann, 1975	Ramamurthiinae Steinmann, 1975
(*Ramamurthia* Steinmann, 1975)	(*Ramamurthia* Steinmann, 1975)
Vandicinae Burr, 1911a	Vandicinae Burr, 1911a
(*Vandex* Burr, 1911a)	(*Vandex* Burr, 1911a)
Strongylopsalinae Burr, 1911a	Strongylopsalinae Burr, 1911a
(*Strongylopsalis* Burr, 1900; *Strongylolabis* Steinmann, 1986a)	(*Strongylopsalis* Burr, 1900; *Strongylolabis* Steinmann, 1986a)
Sparattinae**** Verhoeff, 1902	Sparattinae Verhoeff, 1902
(*Sparatta* Audinet-Serville, 1839; *Auchenomus* Karsch, 1886; *Mecomera* Audinet-Serville, 1839; ***Chaetospania*** Karsch, 1886)	(*Sparatta* Audinet-Serville, 1839; *Auchenomus* Karsch, 1886; *Mecomera* Audinet-Serville, 1839)
Labiinae Burr, 1909	Labiinae Burr, 1909
(*Labia* Leach, 1815; ***Paralabellula*** Kevan, 1997 [in Kevan and Vickery 1997]; *Chaetolabia* Brindle, 1972; ***Paraspania*** Steinmann, 1985; ***Spirolabia*** Steinmann, 1987; *Circolabia* Steinmann, 1987; *Sphingolabis* de Bormans, 1883)	(*Labia* Leach, 1815; ***Apovostox*** Hebard, 1927; *Chaetolabia* Brindle, 1972; ***Chaetospania*** Karsch, 1886; *Circolabia* Steinmann, 1987; *Sphingolabis* de Bormans, 1883)
Geracinae Brindle, 1971b	Geracinae Brindle, 1971b
(*Gerax* Hebard, 1917; *Barygerax* Hebard, 1917; *Eugerax* Hebard, 1917; *Nesolabia* Hincks, 1957 [in Chopard 1957]; *Pseudovostox* Borelli, 1926a; *Yepezia* Brindle, 1982)	(*Gerax* Hebard, 1917; *Barygerax* Hebard, 1917; *Eugerax* Hebard, 1917; *Nesolabia* Hincks, 1957 [in Chopard 1957]; *Pseudovostox* Borelli, 1926a; *Yepezia* Brindle, 1982)
Cosmogeracinae Brindle, 1982	Cosmogeracinae Brindle, 1982
(*Cosmogerax* Hebard, 1933)	(*Cosmogerax* Hebard, 1933)
Caecolabiinae Steinmann, 1990	Caecolabiinae Steinmann, 1990
(*Caecolabia* Brindle, 1975)	(*Caecolabia* Brindle, 1975)
Isopyginae Hincks, 1951	Isopyginae Hincks, 1951
(*Isopyge* Borelli, 1931)	(*Isopyge* Borelli, 1931)
Spongiphorinae Verhoeff, 1902	Spongiphorinae Verhoeff, 1902
(*Spongiphora* Audinet-Serville, 1831; *Filolabia* Steinmann, 1989b; *Formicilabia* Rehn & Hebard, 1917; *Marava* Burr, 1911a; *Pseudomarava* Steinmann, 1989b; *Purex* Burr, 1911a; *Spongovostox* Burr, 1911a; *Vostox* Burr, 1911a; ***Homotages*** Burr, 1909; ***Irdex*** Burr, 1911a)	(*Spongiphora* Audinet-Serville, 1831; *Filolabia* Steinmann, 1989b; *Formicilabia* Rehn & Hebard, 1917; *Marava* Burr, 1911a; *Pseudomarava* Steinmann, 1989b; *Purex* Burr, 1911a; *Spongovostox* Burr, 1911a; *Vostox* Burr, 1911a)
	Homotaginae Srivastava, 1985
	(***Homotages*** Burr, 1909; ***Paratages*** Srivastava, 1987)
	Irdicinae*** Srivastava, 1985
	(***Irdex*** Burr, 1911a)

*Isolaboidinae and *Isolabella* has been removed. See Harz 1975; Sakai 1982; Srivastava 1996b; Kamimura 2025. **Rudracinae has been removed (see the main text). ***Name corrected by Engel and Haas (2007). ****Three tribes are placed: Sparattini Verhoeff, 1902 (*Sparatta*, *Mecomera*); Auchenomini Burr, 1909 (*Auchenomus*); Chaetospaniini*** Steinmann, 1990 (*Chaetospania*).

**Figures 1–8.**
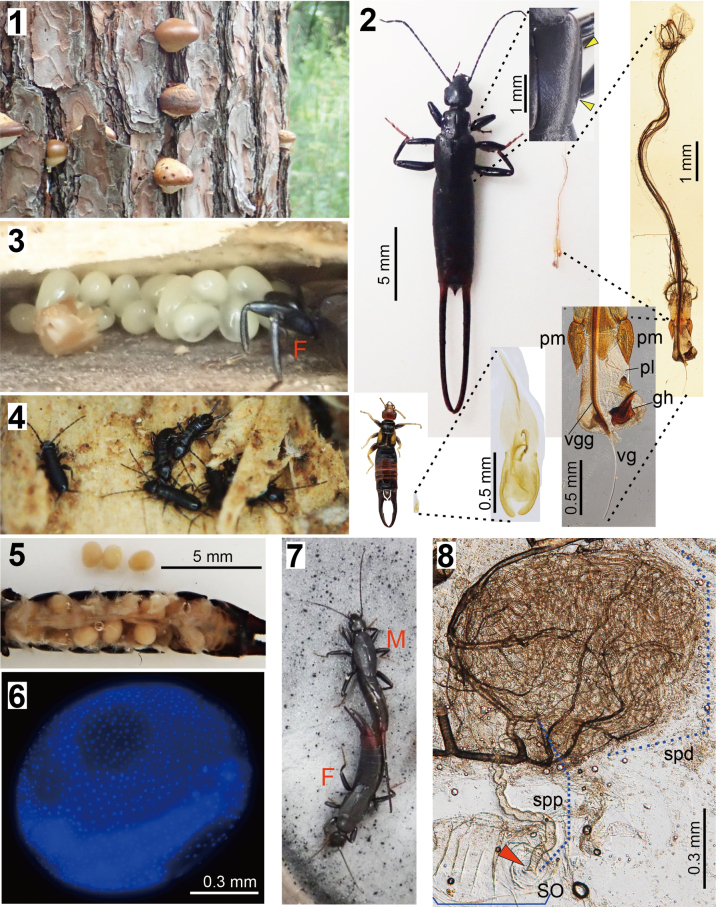
Habitat, habitus, and reproductive traits of *Spongiphora lewisi* (= *Spongovostox lewisi*) compared to *Nesogaster amoenus*.; **1**. Fruiting bodies of *Cryptoporus volvatus* on a pine tree (*Pinus densiflora*); **2**. Male *Sp. lewisi* (right) and *N. amoenus* (left) with their genitalia shown at the same scale. Insets show enlarged views of genital structures and a tegminal keel; **3**. Female *Sp. lewisi* with eggs within 2 days after deposition; **4**. Hatchlings of *Sp. lewisi*; **5**. Female *Sp. lewisi* with the abdominal sternites removed and eggs inside; **6**. Egg in a female ovary of *Sp. lewisi* stained with DAPI; **7**. Mating pair of *Sp. lewisi*; **8**. Spermatheca of *Sp. lewisi*. The yellow and orange arrowheads indicate the tegminal keel and spermathecal opening, respectively. Abbreviations: F, female; gh, genital hook; M, male; pl, penis lobe; pm, paramere; SO, sclerotization around spermathecal opening; spd, distal part of spermatheca; spp, proximal part of spermatheca; vg, virga; vgg, virgal guide.

*Spongiphora lewisi* was originally described by de Bormans (in Burr 1903) from a single male specimen collected in Japan. Since Shiraki (1928), this species has been assigned to Nesogastrinae (Spongiphoridae), based on the presence of lateral keels on the tegmina (forewings), hereafter referred to as tegminal keels. Nesogastrinae is restricted to a single genus *Nesogaster* Verhoef, 1902. The type species of this genus, *N. fruhstorferi* Verhoef, 1902, is considered a junior synonym of *Labia dolicha* Burr, 1897 (= *N. dolichus*). The genus includes more than 20 species distributed across the Great Nicobar Islands (India), Southeast Asia, to Oceania, Australia, and New Zealand (Popham and Brindle 1967; Brindle 1971a; Steinmann 1979, 1989b, 1990; Srivastava 2013). Typically small, shiny, and colorful these earwigs inhabit tropical and subtropical regions (Burr 1908; Brindle 1971a; Steinmann 1990). In contrast, *N. lewisi* expands into the cooler climate of northern Japan (Hokkaido) (Nishikawa 2013; Nishikawa and Yoshitomi 2024). Notably, this species exhibits a robust body exceeding 20 mm in length (including forceps), dull black coloration, and a strong ecological association with the fruiting bodies of *Cryptoporus volvatus* (Harusawa, 1997; Ohmiya, 1998; Nishikawa and Yoshitomi 2024). These traits distinguish *N. lewisi* from other members of the genus *Nesogaster* (hereafter referred to as true *Nesogaster*) (Figs 1, 2; Steinmann 1990; Sakai 1991; Nishikawa and Yoshitomi 2024).

Srivastava (1996a) described *Rudrax brindlei* from Fukien (= Fujian), China, designating it as the type species of the genus *Rudrax* and the subfamily Rudracinae (originally proposed as Rudraxinae, later corrected by Engel and Haas 2007). Rudracinae was established as a monotypic subfamily within Spongiphoridae. However, based on detailed morphological comparisons, including examination of holotypes, Nishikawa and Yoshitomi (2024) recently concluded that *Rudrax brindlei* is conspecific with *Nesogaster lewisi*. As a result, *Rudrax* and Rudracinae are now considered junior synonyms of *Nesogaster* and Nesogastrinae, respectively. This species has been reliably recorded from Japan (reviewed in Nishikawa 2013; Nishikawa and Yoshitomi 2024), China (Srivastava 1996a), Laos (Nishikawa and Yoshitomi 2024), and Vietnam (Steinmann 1983). Records from Taiwan and Korea were rejected by Nishikawa and Yoshitomi (2024).

In the present study, we reassessed the placement of *Spongiphora lewisi* de Bormans, 1903 within Spongiphoridae, based on detailed analyses of male and female genital morphology, reproductive biology, and tegminal keel structure. We also compared molecular data from the DNA barcoding region of this species with those of representative spongiphorids.

## Materials and methods

### Materials examined

The following specimens from the personal collections of Yoshitaka Kamimura [YK], Meliana Gusti [MG], and Masaru Nishikawa [MN] were examined. For *Spongiphora lewisi* de Bormans, 1903, both wild-caught adults and laboratory-reared offspring were studied: 6♂♂ and 4♀♀ (Japan: Yamanashi Prefecture, Fuefuki, ca 1774 m, 4.VI.2025, Y. Kamimura leg., collected from fruiting bodies of *Cryptoporus volvatus* [YK]); 2♂♂ and 2♀♀ (offspring of the two females above, emerged between 1–13.IX.2025 [YK]).

For comparative analysis of external morphology, a male of *Nesogaster amoenus* (Stål, 1855) (Indonesia: West Sumatra Province, Padang Pariaman Regency, Kayu Tanam, 144 m, 23.IV.2025, M. Gusti leg. [MG]) and a male of *Spongovostox sakaii* Nishikawa, 2006 (Japan: Kagoshima Prefecture, Amami-Oshima Island, Sumiyou, 24.V.2018, M. Nishikawa leg. [MN]) were also examined.

For DNA barcode analysis, genomic DNA was extracted, amplified via PCR, and sequenced from the following specimen: *Auchenomus javanus* (de Bormans, 1883), 1♂ (Indonesia: North Sumatra Province, Langkat Regency, Bohorok, 27.VI.2025, M. Gusti leg. [MG]). Although this species has previously been reported from several islands around Sumatra (Mentawai and Enggano Islands under the synonym *Mecomera modiglianii* de Bormans, 1900; Sebesi Island: Borelli 1926b), this constitutes the first confirmed record from the Sumatra mainland.

### Husbandry and reproductive mode

Four wild-caught females of *Spongiphora lewisi* were individually reared in plastic vessels (80 mm diameter × 40 mm height), containing thoroughly moistened sphagnum moss (*Sphagnum* spp.) and three pieces of *Zelkova serrata* bark, which served as harborage and oviposition substrates. The rearing conditions were maintained at 25 ± 1 °C under a 12:12 h light:dark photoperiod. Water, a pellet of commercial cat food (Kin-no-dashi, katsuo-tuna flavor; Inaba-Petfood, Shizuoka, Japan), and a small piece of mushroom (*Pleurotus eryngii*) were provided every 2 days. Oviposition and egg hatching were monitored at the same interval. After all eggs had hatched, the mother was transferred to a new vessel. In some cases, a male reared under identical conditions was introduced to allow mating and sperm replenishment. Nymphs were reared in groups until the third or fourth instar, then transferred to larger plastic containers (150 mm × 200 mm × 80 mm height) lined with plaster of Paris and furnished with bark pieces. They were maintained under the same conditions until adulthood. Imaginal eclosion was checked every 2 days, and newly emerged adults were isolated in individual vessels.

Two deceased females were preserved in 70% ethanol, and dissected in phosphate buffered saline (PBS) under a stereomicroscope (SZX16; Olympus Tokyo, Japan) using fine forceps. Ovarian eggs were extracted, rinsed twice in PBS, and stained with 2 μg/mL 4’,6-diamidino-2-phenylindole (DAPI) in PBS for 24 h at 4 °C. After a final PBS rinse, the eggs were mounted on APS-coated glass slides (S8112; Matsunami Glass, Osaka, Japan) using 2% carboxymethyl cellulose. Fluorescence was observed using a BZ-X800 fluorescence microscope (Keyence, Osaka, Japan) equipped with a × 4 objective lens and a blue fluorescence filter set (excitation: 395 nm; dichroic mirror: > 425 nm; absorption filter: > 460 nm). Fully-focused, deconvoluted images were generated using the microscope’s sectioning module and analysis software.

### Observation of courtship and mating

Virgin males and virgin females, 6–10 days post-imaginal eclosion, were paired in plastic vessels (65 mm diameter × 95 mm height) with plaster of Paris at the base (*n* = 2 pairs). Behaviors were recorded using a time‐lapse camera (GZ‐MG980S; Victor Company of Japan, Yokohama, Japan) at a rate of one frame every 2 s for 24 h. Each trial began at 13:00 and ended at 13:00 the following day, with the 20:00–08:00 interval conducted in occurring in darkness under dim red light. To allow acclimation, females were introduced into the vessel 10 min prior to the start of recording: males were added immediately before the trial commenced. Copulation durations were measured from the video recordings to the nearest minute. Following the mating trials, females were maintained under the same conditions as wild-caught individuals of the parent generation.

### Female and male genital morphology

Male and female genital structures of *Sp. lewisi* were dissected in PBS under the stereomicroscope, and examined using a differential interference contrast microscope (BX53; Olympus) at × 100–400 magnification. Images were captured with an Olympus Pen e-pl1s digital camera. For *N. amoenus*, male genitalia were dissected under a SMZ1270 stereomicroscope (Nikon, Tokyo, Japan) and documented using an EOS R10 (Canon, Tokyo, Japan). Multi-focused montage images were generated using Helicon Focus Pro. (Helicon Soft Ltd., http://www.heliconsoft.com/). Artifacts, ghosting, and extraneous elements (e.g., unfocused parts or surrounding tissue) were removed using the software’s retouching function. Final adjustments to color balance, contrast, and sharpness were performed in Adobe Photoshop CS6.

### Tegminal keels

The tegminal keels of *Sp. lewisi* and *Sv. sakaii* were observed under the SZX16 stereomicroscope. Composite images of intact specimens were also obtained using the microscope mode and focus-stacking sub-mode of a Tough-TG5 digital camera (Olympus). For *N. amoenus* imaging was conducted as for the genitalia.

For male samples of *Sp. lewisi* and *N. amoenus* (*n* = 1 each), the tegmina were cut at the center using fine ophthalmic scissors to observe their cross-sections. The posterior portions of the tegmina were dried and examined by scanning electron microscopy (SEM: JSM-6510; JEOL Ltd., Tokyo, Japan) at an acceleration voltage of 10 kV, following gold coating by ion sputtering (Hitachi E101; Hitachi Ltd., Tokyo, Japan).

### Molecular data analysis

The DNA barcodes, i.e., the sequences (658 base pairs) of the mitochondrial cytochrome c oxidase subunit I (COX1) gene amplified by the universal primer set (LCO1490 5’-ggtcaacaaatcataaagatattgg-3’ and HCO2198 5’-taaacttcagggtgaccaaaaaatca-3’: Folmer et al. 1994), were retrieved from the DNA Data Bank of Japan (DDBJ) for the following nine species of Spongiphoridae (sequenced in Kamimura et al. 2023a, 2023b): *Sp. lewisi* (registered as *Nesogaster lewisi*) [accession no. LC715986], *Spongovostox sakaii* Nishikawa, 2006 (Spongiphorinae) [LC767844], *Marava arachidis* (Yersin, 1860) (Spongiphorinae) [LC715968], *Labia minor* (Linnaeus, 1758) (Labiinae) [LC767866], *Paralabellula curvicauda* (Motschulsky, 1863) (Labiinae) [LC715972], *Chaetospania javana* Borelli, 1926b (Sparattinae) [LC715967], *Pseudovostox brindlei* Srivastava, 2003a (Geracinae) [LC715969], and *N. amoenus* (Nesogastrinae) [LC715977].

For *Auchenomus javanus* (Sparattinae), the DNA barcode was newly sequenced. DNA was extracted from a leg after washing with 500 μL TE solution (pH 8.0). The cleaned legs were placed on a sterilized disposable plate and transferred to 105 μL solvent solution, consisting of 100 μL Chelex-TE solution and 5 μL Qiagen proteinase K. The mixture was incubated at 56 °C for 24 h, followed by heating at 99 °C for 10 min to inactivate the enzyme. Each PCR reaction contained 5 μL 2 × PCR Buffer, 2 μL 2 mM dNTPS, 0.3 μL of forward primer (10 μM), 0.3 μL reverse primer (10 μM), 1.7 μL distilled water, 0.2 μL KOD FXneo DNA polymerase (1.0 unit/μL; Toyobo, Osaka, Japan), and 1 μl DNA template. PCR amplification was carried out using a MiniAmp Thermal Cycler (Thermo Fisher Scientific, U.S.) with the cycling condition of one cycle of 94 °C for 2 min, 35 cycles of 94 °C for 15 s, 51 °C for 15 s, 72 °C for 15 s, and final annealing at 72 °C for 6 min for one cycle, and holding at 4 °C indefinitely. Amplified products were confirmed using a 2.4% agarose gel. The ExoSAP process was carried out by adding 2 μL ExoSAP solution (0.1 μL of ExoSAP and 1.9 μL distilled water) to the 8-connected tube containing 5 μL PCR product, and incubated at 37 °C for 4 min, followed by 80 °C for 1 min. Sequencing was performed by FASMAC (Tokyo, Japan). Chromatograms were visually inspected and manually edited if necessary. After removing the primer sequences and parts of low quality, the COI sequence (414 bp) was deposited in DDBJ/ENA/GenBank [Accession Number PX831130]. Calculation of interspecific *p*-distances was performed with MEGA version 12.0.14 (Kumar et al. 2024).

### Taxonomic and terminology systems followed

We follow Steinmann’s (1990) system for the classification of Spongiphoridae (Table 1). The terminology used for the dermapteran wing, female genitalia, and male genitalia largely follows conventions established by Haas and Kukalová-Peck (2001), Schneider and Klass (2013), and Kamimura (2025), respectively.

## Results

### Reproductive biology

Wild-caught females laid one or two clutches under laboratory conditions. Dermapteran eggs are generally a uniform cream color just after oviposition, later differentiating into a cream-colored embryo section and a more transparent surrounding area as development progresses (Shimizu and Machida 2024). By contrast, in *Sp. lewisi* even eggs within two days of deposition already exhibited a two-colored state, indicating advanced development (Fig. 3). The mother remained close to the egg mass, providing protection, as generally observed in earwigs (Costa 2006; Shimizu and Machida 2024).

Hatching of the nymphs was observed at between 4 and 6 days after oviposition was confirmed (Fig. 4). In dead females, large, developed eggs—up to 28—were observed in the abdomen, aligned along the elongated lateral oviducts (Fig. 5).

Fluorescence microscopy of DAPI-stained eggs confirmed that embryonic development had already begun within the ovaries (*n* = 2: Fig. 6). Shimizu and Machida (2024) recognized nine stages in the dermapteran embryonic development. In a female that died at 18 days (including 7-day cohabitation with a male) after hatching of the previous clutch, the long diameter of ovarian eggs was ~ 1.25 mm. These reached Stage 3 of Shimizu and Machida (2024), as evidenced by the blastoderm differentiated into two areas with different cellular densities. In another female that died at 16 days (including 7-day cohabitation with a male) after hatching of the previous clutch, ovarian eggs were nearly the same size but showed a further developed, elongate embryo along the egg surface (Stage 4; Fig. 6).

New adults began to emerge at ~ 60 days after hatching at 25 °C. When virgin males and females cohabited in a container, mating occurred readily (Fig. 7). In two pairs of observations, the numbers of matings per 24 h were 6 and 19, with average mating durations of 18 min (range 4–32) and 13 min (range 2–36), respectively. In some cases, upon encountering the female, the male used its long forceps to gently grasp the female’s body, engaging in grooming-like behavior. However, no other characteristic courtship behaviors were observed. One female laid eggs at 23–24 days after mating (19 times of genital couplings); the eggs, at the time of discovery, were distinctly divided into two differently colored regions, indicating that embryonic development had already progressed.

### Female and male genitalia

The male genitalia of *Sp. lewisi* are conspicuously elongate (e.g., Nishikawa and Yoshitomi 2024); in our samples, they reached ~ 50% of the body length excluding forceps (Fig. 2). Within the membranous penis lobe, a hook-like, large, conspicuous sclerite—termed the genital hook hereafter—and an elongate sclerite along with a thin virga—termed the virgal guide—are enclosed (Fig. 2). These structures were also illustrated in previous studies (Srivastava 1996a; Nishikawa and Yoshitomi 2024).

The spermatheca of *Sp. lewisi* is illustrated in Fig. 8 for the first time. The area surrounding the spermathecal opening was weakly sclerotized and exhibited a wrinkled pattern. The spermatheca itself consists of a single tube, divided into two sections: a coiled, spiral-shaped basal portion and a longer, narrower distal portion.

### Tegminal keels

**Figures 9–11.**
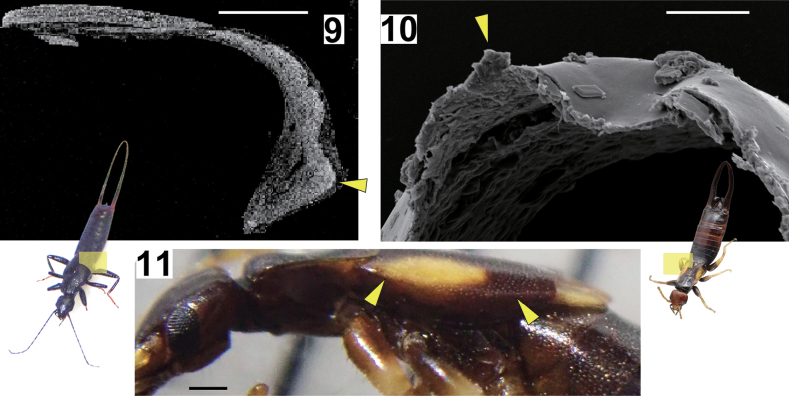
Tegmina (**9–11**) and their cross-section SEM micrographs with schematics of the sectioning plane (**9, 10**) of *Nesogaster amoenus* (**9**), *Spongiphora lewisi* (= *Spongovostox lewisi*) (**10**), and *Spongovostox sakaii* (**11**). The yellow arrowheads indicate the tegminal keels. Scale bars: 0.3 mm (**9, 11**); 0.03 mm (**10**).

No structural differences were observed in the tegminal keels between *N. amoenus* and *Sp. lewisi*: in both cases, the keel was formed by thickening of the outer tissue (Figs 9, 10). However, the direction of keel protrusion differed. The tegminal keel of *Sp. lewisi* extended laterally (Fig. 9), while in *N. amoenus* it extended dorsally (Fig. 10). Although the members of the genus *Spongovostox* have been considered not to possess keels in the tegmina, observations of *Sv. sakaii* revealed a keel (or fold) that is not very pronounced, directed laterally—similar to that observed in *Sp. lewisi* (Fig. 11).

### Comparison of DNA barcoding region

Comparisons of the DNA barcodes revealed that *Sp. lewisi* shows the highest affinity to two species of Spongiphorinae, particularly to *Sv. sakaii*, among representatives of five subfamilies of Spongiphoridae (Table 2). Although the extremely high evolutionary rate of this gene region makes detailed comparisons difficult, the *p*-distance between *N. amoenus* (Nesogastrinae) and *Sp. lewisi* (0.2568) was one of the largest among those examined (Table 2).

**Table 2.** Percent divergence (p-distance) between the sequences of Spongiphoridae. Intra-subfamilial comparisons are shown in bold.

Subfamily	Species	1: SL	2: SS	3:MA	4: AJ	5: LM	6: PC	7: CJ	8: PB
In question	1: *Spongiphora lewisi* (SL)								
Spongiphorinae	2: *Spongovostox sakaii* (SS)	0.1383							
Spongiphorinae	3: *Marava arachidis* (MA)	0.1626	0.1505						
Sparattinae	4: *Auchenomus javanus* (AJ)	0.1763	0.1908	0.2029					
Labiinae	5: *Labia minor* (LM)	0.1945	0.1945	0.2097	0.2246				
Labiinae	6: *Paralabellula curvicauda* (PC)	0.1960	0.1930	0.1991	0.2271	0.2128			
Sparattinae	7: *Chaetospania javana* (CJ)	0.1976	0.1793	0.1976	0.2391	0.2112	0.2112		
Geracinae	8: *Pseudovostox brindlei* (PB)	0.2036	0.1824	0.1884	0.2536	0.2447	0.2158	0.2401	
Nesogastrinae	9: *Nesogaster amoenus* (NA)	0.2568	0.2675	0.2796	0.2899	0.2416	0.2508	0.2690	0.2888

### Taxonomy

Based on the evidence outlined above, we propose reinstatement of the combination *Spongovostox lewisi* (de Bormans, 1903), proposed by Sakai (1970b).

#### Family Spongiphoridae Verhoeff, 1902

**Subfamily Spongiphorinae Verhoeff, 1902**.

**Genus *Spongovostox* Burr, 1911b (type species: *Forficula quadrimaculata* Stål, 1855)**.

##### Spongovostox lewisi

Taxon classificationanimaliaDermapteraSpongiphoridae

(de Bormans, 1903)

F290E6D4-B614-5102-AF81-08541A12C193

Spongiphora lewisi de Bormans, 1903: in Burr (1903): 234 (♂ holotype: now in Natural History Museum, London); type locality: Hako, Japan (see Nishikawa 2009 for discussion on the type locality).
Labidurodes singularis Shiraki, 1906: 8 (type ♂, ♀: ♂ type now in National Taiwan University); type locality: Sapporo, Hokkaido, Japan (synonymized with Nesogaster lewisi by Ōkuni 1913: 184 and also by Steinmann 1989b: 406).
Labidurodes nigritus Shiraki, 1907: 91, fig. 1 (type ♂, ♀: ♂ type now in National Taiwan University); type locality: Jozankei, Hokkaido, Japan (synonymized with Nesogaster lewisi by Sakai, 1990: 184, with wrong spelling nigrita).
Labidurodes ? singularis: Burr 1911b: 32.
Labidurodes ? nigritus: Burr 1911b: 32.
Spongovostox ? lewisi: Burr 1911b: 52.
Nesogaster lewisi : Shiraki 1928: 14.
Nesogaster nigritus : Shiraki 1928: 14.
Nesogaster nigrita : Popham and Brindle 1967: 36.
Anisolabis ? singularis: Sakai 1970a: 66.
Spongovostox lewisi : Sakai 1970b: 63.
Rudrax brindlei Srivastava, 1996a: 77, figs 1–7 (♂ holotype: now in Bishop Museum, Honolulu, Hawaii; type-locality: South China (Fukien, Changting, Niuling) (synonymized with Nesogaster lewisi by Nishikawa and Yoshitomi 2024: 322).

## Discussion

### *Generic and subfamilial placements of* Spongiphora lewisi

Although the tegminal keels of *Spongiphora lewisi* and *Nesogaster amoenus* have essentially similar structures (conspicuous thickening of the outer-surface tissues at the keels), the following four characteristics of *Sp. lewisi* indicate its close affinity to *Spongovostox* spp., particularly ovoviviparous species with elongate male genitalia.

First, among Eudermaptera, elongate male genital structures with both a conspicuous genital hook and virgal guide have been reported for *Marava arachidis* and *Spongovostox semiflavus* (de Bormans, 1894) (Kamimura et al. 2016b; Kamimura and Lee 2017). To the best of our knowledge, no similar structures have been reported for true *Nesogaster* spp. The images in Nishikawa (2006) imply that male *Sv. sakaii* also possesses these structures although the genitalia are not as elongate.

Second, the spermathecal structures of *Sp. lewisi* exhibit characteristics unique to spongiphorine species. In Spongiphoridae, a spermatheca lacking a sclerotized capsule at the distal end has been reported only in species of Spongiphorinae: *M. arachidis* (Schneider and Klass 2013; Kamimura et al. 2016b), *Sv. semiflavus* (Schneider and Klass 2013), and *Sv. mucronatus* (Stål, 1860) (Kamimura et al. 2016a). An exception is *Pseudovostox brindlei* (Geracinae: Kamimura et al. 2016a), but in this species, the spermathecal opening region lacks conspicuous sclerotization with fingerprint-like or wrinkled patterns. Extensive spiral coiling in the basal region is also common between *Sv. mucronatus* and *Sp. lewisi*. By contrast, the spermathecae of true *Nesogaster* spp. (*N. halli* Hincks, 1949 in Hudson 1973 and *N. amoenus* in Kamimura et al. 2016a) are less elongate and possess a sclerotized capsule at the distal end, as seen in many other spongiphorids. Notably, the genital hook of *M. arachidis* penetrates the female body near the spermathecal opening during copulation, leaving a wound scar (Kamimura et al. 2016b). No sign of such copulatory wounding was detected in *Sp. lewisi* as in the case of *Sv. semiflavus* (Kamimura and Lee 2017).

Third, developing eggs in ovaries, indicating fertilization at that site, have been reported for four spongiphorid species across three subfamilies: *Chaetospania borneensis* (Dubrony, 1879) (Sparattinae: Kočárek 2009), *Sphingolabis hawaiiensis* (de Bormans, 1882) (Labiinae: Marrucci 1955; Matzke 2011), and two spongiphorine species *M. arachidis* and *Sv. semiflavus*. In *M. arachidis*, *Sv. semiflavus*, and *Sl. hawaiiensis*, females lay eggs just before hatching, with embryos already showing pigmented eyes (Herter 1943; Patel and Habib 1978; Matzke 2011; Kamimura et al. 2016b; Kamimura and Lee 2017). These species are thus nearly ovoviviparous. In comparison, deposited eggs of *Sp. lewisi* required an additional 4–6 days of maternal care before hatching. This aligns with the observation that newly deposited eggs of this species lack discernible eyes, limbs, and other nymphal organs (Fig. 2).

Two types of ovarian morphology have been reported in Spongiphoridae (Biliński et al. 2014): longer ovarioles attached to shorter lateral oviducts in *Irdex chapmani* Brindle, 1980 (Spongiphorinae), and shorter ovarioles attached to longer lateral oviducts (Eudermaptera in general). The ovaries of *Sp. lewisi* were classified as the second type, typical of Spongiphoridae. Shimizu and Machida (2024) reported that eggs of eudermapteran species of similar body size to *Sp. lewisi*, e.g., *Proreus simulans* (Stål, 1860) (Chelisochidae) and *Forficula scudderi* de Bormans, 1880 (Forficulidae), require ~ 10 days to hatch at 23–25 °C. The extraordinarily short incubation period after oviposition in *Sp. lewisi* is consistent with the view that females lay eggs in the mid-stage of embryogenesis. In dermapteran species, maternal stress can sometimes lead to cessation of egg protection, so egg counting was not performed in this study. However, Ohmiya (1998) reported 18 eggs in a clutch of *Sp. lewisi*. This relatively small clutch size is likely related to the substantial development of eggs within the ovary. Kamimura et al. (2016b) experimentally demonstrated that sperm of *M. arachidis* travel to the ovaries for fertilization after being stored in the tubular spermatheca. A similar mechanism of ovarian fertilization likely functions in *Sp. lewisi*.

Finally, our comparison of DNA barcodes revealed the closest affinity of *Sp. lewisi* to species of Spongiphorinae. All of the evidence outlined above strongly supports the view that *Sp. lewisi* does not belong to *Nesogaster*, but is a member of *Spongovostox*. If we disregard its tegminal keels, this species aligns well with *Spongovostox* based on contemporary keys of Spongiphoridae (Steinmann 1990; Srivastava 1996a). For unknown reasons, Burr (1911b) and Sakai (1970b) previously placed this species under *Spongovostox*, with or without a question mark. Also considering the slender and simple tarsal segments (vs broader and notably hairy in *Spongiphora*) and the normal antennal segments (vs notably conical in *Marava*) (Steinmann 1990), this treatment is reinstated here.

Given the placement of de Bormans’s (1903) *lewisi* in *Spongovostox*, the present study also provides insights into the diversity of reproductive biology among members of this genus and Spongiphorinae. In *M. arachidis* (Herter 1943; Kamimura et al. 2016b) and *Pseudomarava prominens* Steinmann, 1990 (Briceño and Eberhard 1995: as *Pseudomarava prominensis*), males grasp the antennae of female mates with their mouthparts to initiate genital coupling. A snapshot of a mating pair of *Sv. sakaii* also implies the occurrence of this behavior, which has been reported only for Spongiphorinae among Dermaptera (Kim and Nishikawa 2017). However, this type of mating has not been detected in *Sv. lewisi*, as in the case of *Sv. semiflavus* (Kamimura and Lee 2017).

### Tegminal keels as a possible convergent trait

This study demonstrates that an integrative approach, including laboratory rearing, behavioral observations, and examination of development, can contribute to the stabilization of dermapteran classification. Our approach also revealed that the following two characteristics likely resulted from convergent evolution between *Sv. lewisi* and true *Nesogaster* species: association with fruiting bodies of fungi and tegminal keels. *Spongovostox lewisi* is strongly associated with fungi: most specimens of this species in Japan were collected from the fruiting bodies of *Cryptoporus volvatus* (see Introduction). Among true *Nesogaster* species, *N. amoenus* are frequently found on soft or hard mushrooms on dead logs in Southeast Asian countries (Kamimura et al. in press; YK, unpublished data). However, the association is not as strong as in the case of *Sv. lewisi*, and a similar fungal preference has also been reported for *Sv. sakaii* (Kim and Nishikawa 2017).

Although the cross-sectional profiles are essentially similar between the tegminal keels of *Sv. lewisi* and *N. amoenus*, they are oriented in different directions. It is reasonable to consider that these keels serve as structural reinforcements to prevent the wings from bending under pressure from air or physical contact, particularly during flight or when maneuvering through narrow spaces. Given this functional significance, it is likely that tegminal keels have evolved multiple times within Dermaptera. It is notable that we discovered a keel or fold in the horizontal direction—although not prominent—in another species of *Spongovostox*. The distribution of this trait within the genus *Spongovostox* remains to be investigated in future studies. Species with larger body sizes, such as *Sv. lewisi*, would experience greater air resistance and gravitational forces during flight, necessitating stronger wing structures and thus well-developed tegminal keels. It is thus likely that the keel of *Sv. lewisi* is conspicuous, combined with its dorsoventrally flattened body shape. The presence or absence of tegminal keels is used as a diagnostic trait for generic classifications in many dermapteran families (e.g., Steinmann 1986b, 1989a, 1990, 1993; Srivastava 1988, 2003b, 2013). The present study, however, illustrates that reliance on this single trait can result in instability of classification. Possible confounding effects of convergent evolution in other dermapteran taxa should be tested in future studies using molecular data.

### Subfamilial classification of Spongiphoridae

Based on the discussion given above, a revised key is proposed for the subfamilies of Spongiphoridae.

### A revised key for subfamilies of Spongiphoridae (Based on males: modified from Steinmann 1990)

**Table:** 

1	Lateral margins of tegmina with a well-developed vertical keel or ridge that forms a lateral explanate rim to the dorsal surface	**2**
–	Lateral margins of tegmina without a longitudinal keel or ridge. If keels present, not like a lateral rim to the dorsal surface, but projecting to the lateral side	**6**
2	Tarsal segments elongate and thin; basal segment ~ 5 × as long as wide. Tegmina rough, granular, hairy	**Pericominae (*Pericomus* , *Parapericomus* )**
–	Tarsal segments shorter and thicker; basal segment at most 3–4 × as long as wide. Tegmina smooth, or nearly smooth, not or only slightly granular or roughened	**3**
3	Antennal segments coniform; apically slightly constricted; apices of each slightly, although perceptibly narrowed	**4**
–	Antennal segments cylindrical	**5**
4	Forceps pincer shaped. Pygidium lamelliform of various shapes. Virga narrower than width of paramere	**Nesogastrinae (*Nesogaster* )**
–	Forceps nearly straight, not pincer shaped. Pygidium slightly angular. Parameres thin and long; narrower than virga	**Ramamurthinae (*Ramamurthia* )**
5	Number of antennal segments 16–20. Tegmina normally developed, posterior margin truncate or slightly arcuately emarginate. Forceps elongate, weakly arcuate, curved	**Vandicinae (*Vandex* )**
–	Number of antennal segments 12–15. Tegmina abbreviated, shortened, or squamiform; posterior margin never truncate. Forceps shorter, apically more strongly inclinate	**Strongylopsalinae (*Strongylopsalis* , *Strongylolabis* )**
6	Body dorso-ventrally strongly compressed, flattened	**Sparattinae (Auchenomini: *Auchenomus* ; Sparattini: *Sparatta* , *Mecomera* ; Chaetospaniini: *Chaetospania* )**
–	Body cylindrical in cross-section, not strongly flattened dorso-ventrally	**7**
7	Tarsal arolia between claws present	**Geracinae (*Gerax* , *Eugerax* , *Barygerax* , *Nesolabia* , *Pseudovostox* , *Yepezia* )**
–	Tarsal arolia between claws absent	**8**
8	Ultimate abdominal tergite semicircular and sloping down to the pygidium. Most of abdominal tergites 5–9 hidden beneath the proceeding tergites	**Cosmogeracinae (*Cosmogerax* )**
–	Ultimate abdominal tergite rectangular, not sloping down to the pygidium. Abdominal tergites 5–9 less hidden	**9**
9	Head without eyes, blind species	**Caecolabiinae (*Caecolabia* )**
–	Head with well-developed eyes	**10**
10	Eyes large, as long as or longer than length of first antennal segment	**11**
–	Eyes small, shorter than length of first antennal segment	**Labiinae (*Labia* , *Paralabellula* , *Circolabia* , *Spirolabia* , *Paraspania* , *Chaetolabia* , *Sphingolabis* )**
11	Forceps short, about as long as width of ultimate tergite, their bases wide, apically strongly curved	**Isopyginae (*Isopyge* )**
–	Forceps perceptively longer than width of ultimate tergite	**Spongiphorinae (*Spongiphora* , *Purex* , *Irdex* , *Formicilabia* , *Vostox* , *Filolabia* , *Spongovostox* , *Homotages* , *Marava* , *Pseudomarava* )**

## Supplementary Material

XML Treatment for Spongovostox lewisi

## Additional information

### Use of AI

No use of AI was reported.

### Author ORCIDs

Yoshitaka Kamimura https://orcid.org/0000-0003-2882-491X

Meliana Gusti https://orcid.org/0000-0001-6936-4089

### Data availability

All of the data that support the findings of this study are available in the main text.
